# Migraine and Stroke: A Scoping Review

**DOI:** 10.3390/jcm13185380

**Published:** 2024-09-11

**Authors:** Neal Nathan, Angeline Ngo, Suzan Khoromi

**Affiliations:** Department of Neurosciences, University of California-San Diego, 200 W Arbor Drive, San Diego, CA 92103, USA; nnathan@health.ucsd.edu (N.N.);

**Keywords:** migraine, stroke, migrainous infarction, cortical spreading depression

## Abstract

An increased risk of ischemic stroke in migraine with aura (MA) has been consistently demonstrated. The pathophysiology of risk factors is not yet well understood. Several mechanisms have been proposed to explain the association between MA and ischemic stroke including decreased focal cerebral blood flow and other phenomena linked with cortical spreading depression (CSD) as well as neurovascular pathology, which appear to play a key role in MA. In addition to genetic predisposition, other classic stroke risk factors such as atrial fibrillation, emboli, migraine-associated vasculopathy, endothelial dysfunction, platelet dysfunction, coagulation pathway abnormalities, and inflammatory factors have been examined and investigated. For further clarification, distinctions have been made between features of migrainous infarctions and non-migrainous infarctions among migraineurs. Furthermore, the association is less clear when considering the mixed results studying the risk of ischemic stroke in migraines without aura (MO) and the risk of hemorrhagic stroke in people with all types of migraine. Translational research is investigating the role of biomarkers which can help identify vascular links between stroke and migraine and lead to further treatment developments. We performed a scoping review of the PubMed database to further characterize and update the clinical connections between migraine and stroke.

## 1. Introduction

Migraine is a complex neurological disease with widespread prevalence and associated disability. The Global Burden of Disease Study in 2021 estimated that 1.16 billion people endure migraines. Migraine remains an enigmatic phenomenon that can cause striking but reversible neurological symptoms. It is one of the leading neurological causes of disability in adults aged between 20 and 59 years old, only outpaced by stroke [1]. Therefore, because of the global impact of migraine and its potential in resulting in stroke, as a rare condition of migrainous infarction, it is important to review the commonalities between migraine and stroke and the pathophysiological features of migraine that may increase the risk for stroke.

Myriad theories describe the various connections between migraine and stroke. The aim of this review is to shed light on the associations between these two clinical entities and the pathophysiological features of migraine that may lead to stroke through a critical evaluation of the literature and by discussing current models of pathogenesis, updated risk factors, treatment considerations, and future developments in migraine and stroke.

## 2. Methods

**Figure 1:**
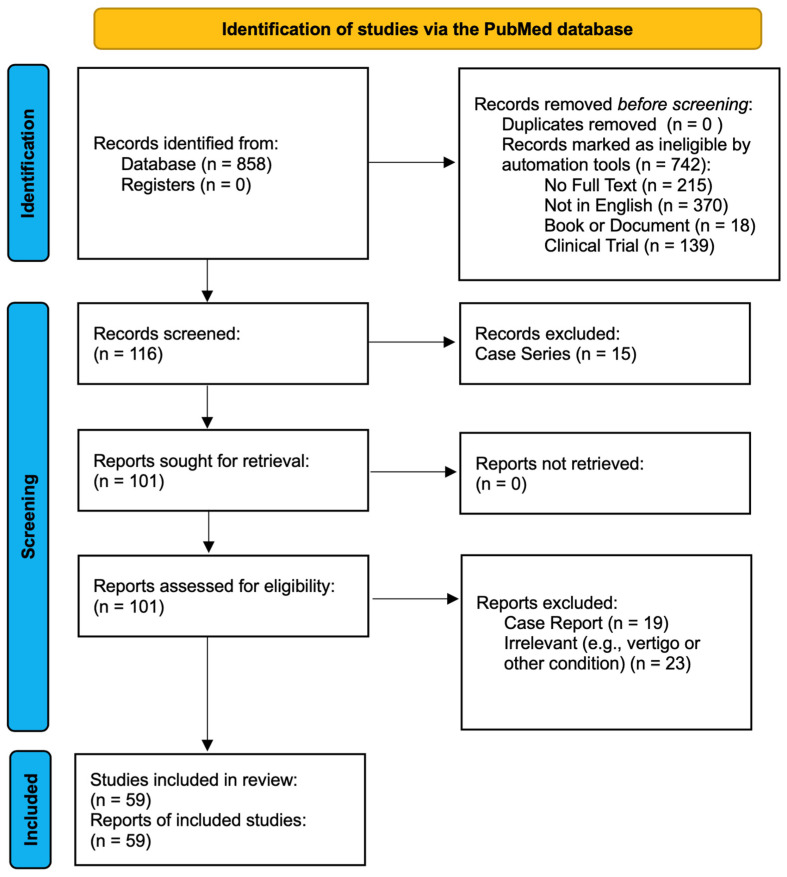
PRISMA flow diagram for scoping review.

The search strategy was informed by the principles of Preferred Reporting Items for Systematic Review and Meta-Analysis (PRISMA) guidelines for scoping reviews. A literature search was performed utilizing the PubMed database, and was last performed on 15 July 2024. Initial search terms were “migraine” and “stroke” with a filter of “review”. Informed by this initial search, additional search terms for each sub-topic such as cortical spreading depression, microembolism, and cerebral hypoperfusion were added in separate searches. To enable a comprehensive review, no geographic or date restrictions were imposed. Only full texts in the English language were included. The range in years of articles available for review in the PubMed database was 1976 to 2024. Only peer-reviewed publications were chosen, and other forms of publications such as books and clinical trials were excluded. Population-level clinical surveys were prioritized. When possible, large studies that pooled multiple data sets, or meta-analyses, were selected. Unless highlighting a pathophysiological phenomenon, case reports were not included. Data charting was carried out independently in a shared document to avoid duplication, and variables selected were both conclusions of research studies and often the numerical results of an odds ratio or relative risk calculation, when possible, to demonstrate relevant findings. The synthesis of results occurred in a narrative format in subsections below to discuss key conclusions of the extant literature. Figure 1 demonstrates the search methodology and results, which are further discussed.

## 3. Theory of Migraine

The neurovascular hypothesis of migraine dates back as early as the 17th century to Thomas Willis. The neural component, i.e., cortical spreading depression (CSD), is now believed to be a key phenomenon in the pathophysiology of migraine aura, and the activation of the trigeminovascular system, on the other hand, appears to be responsible for the painful phase of migraine, in part due to the release of pro-inflammatory peptides centrally and peripherally [2]. The latter is likely accompanied by the peripheral vasodilation of meningeal vessels and is contributory to pain. The mechanism of migraine has yet to be fully elucidated, but the emergence of a neural model of migraine highlights the dynamic complexity of migraine phenomena. The hypothesis of CSD has arisen out of animal models and is thought to be the pathophysiological basis of migraine aura. Cortical spreading depression is a depolarizing, electrochemical wave that propagates through gray matter, resulting in changes in synaptic activity, extracellular ion concentrations, blood flow, and metabolism [2]. One of the ways it is thought to generate migraine pain is by activating the trigeminal nucleus caudalis [3]. 

Cortical spreading depression is a highly complex phenomenon that was introduced by Leao and theorized to be a process akin to “cerebral circulatory arrest” [4]. It is characterized by neuronal and glial depolarizations which spread across the cortex at a rate of approximately 2 to 5 mm/min. This phenomenon has been best described in the occipital lobe but can occur in other parts of the cortex and accounts for visual, sensory, motor, and language auras in migraine. The first animal studies documenting cortical spreading depression were performed on rabbits in 1944 [4]. These findings were further substantiated by imaging studies including Xenon-133 inhalation and emission tomography measuring regional cerebral blood flow during migraine attacks, demonstrating a 12 to 17% decrease in regional cerebral blood flow, and more recently, functional magnetic resonance imaging studies have shown that during MA, subjects experience a decrease in focal cerebral blood oxygen level and oligemia which mirrors the temporal progression of cortical spreading depression in animal models [5,6,7]. This process has been postulated as a risk factor for ischemic stroke in migraine with aura (MA), and although the level of hypoperfusion alone remains above the ranges required for frank ischemia, it most likely can be a strong contributory factor to stroke among otherwise susceptible individuals [8,9]. 

Studies have shown that vasodilation does occur during CSD and disrupts neurovascular coupling as well [3]. In many instances, CSD appears to originate in the occipital lobe, i.e., the territory of the posterior cerebral artery (PCA). Therefore, it is not surprising that visual aura is the most common aura experienced by patients with MA. Interestingly, the PCA territory appears to be a predominant site of stroke in migraine patients: in several small series, over 70% of young migraine patients with migrainous stroke were found to have lesions in the territory of the posterior cerebral circulation, and many other studies have reported PCA hypoperfusion in the context of migraine with aura, so clearly, focal oligemia can be a culprit in stroke in the setting of migraine with aura [10]. There are various postulations as to why the PCA territory is susceptible to ischemic stroke in the context of migraine including the common fetal-type PCA variant that leads to an incomplete Circle of Willis, microemboli through a vertebral artery pseudoaneurysm, and less robust secondary vascular symptoms of arterioles and capillaries compared to other vascular territories [11,12,13]. One consideration when evaluating the reported strong link between migraine with aura and the PCA territory is the possibility of over-reporting, as visual auras are the most common form of aura, and since visual auras involve the PCA territory, there can be selection bias in comparing the connection between the PCA and migraine compared to other vascular territories and migraine. 

Other phenomena accompany CSD such as efflux of excitatory neurotransmitters and changes in mediators of inflammation, growth factors, and endothelial function. Astrocytes, a glial cell, are involved in regulation neurotransmitters and ion levels, and are therefore implicated in CSD. Therefore, genetic alterations and metabolic disruptions such as fasting, which perturb astrocyte function, increase the risk of migraines [14,15]. Beyond neuronal modifiers, regulators of cerebral vasculature are also involved in migraine. Many studies have aimed to identify the basis for endothelial dysfunction which may be present among patients with MA. Increases in C reactive protein (CRP) and cytokines such as tumor necrosis factor-alpha, IL-1B, IL-6, and IL-10 have been reported in migraine patients [16,17]. However, there are mixed data for CRP and other inflammatory markers including cytokines, with small studies reporting trends toward a positive correlation between CRP and migraine with aura, but an insignificant *p*-value of 0.08 [16]. MA may be associated with an increased level of circulating endothelial microparticles, potentially contributing to endothelial dysfunction [18]. Migraineurs have been found to have increased levels of biomarkers of endothelial activation. This supports the notion that blood vessels play a role in the mediation of migraine pathophysiology. Furthermore, changes to vascular structure have been observed in people with migraine as ultrasound studies have demonstrated that carotid intima media thickness is increased in people with migraine compared to controls [19]. There may even be a proportional pattern to this increase in intima thickness as this structural aberration was more pronounced in people who experience chronic migraines compared to those who have episodic migraines [19]. Interestingly, those with chronic migraines had less vasodilatory reactivity compared to those who suffer from episodic migraines, which may be attributable to the greater increase in the intima and media thickness, as these layers are what respond to vasodilatory agents like nitric oxide [19].

Another proposed pathophysiologic mechanism of migraine is glymphatic system dysfunction secondary to the vasoactive and electrochemical gradient aberrations triggered by cortical spreading depression [20]. Thus, CSD can perpetuate migraines as compounds that can further provoke migraines are not filtered out if cerebrospinal fluid is not flowing properly. One study utilizing an animal model of migraine by inducing CSD found that CSD alters 11% of the CSF proteome, with the up-regulation of proteins that directly activate receptors in the trigeminal ganglion, including calcitonin gene-related peptide (CGRP), a pathogenic protein that triggers migraines [21]. Thus, disruptions in the flow or absorption of cerebrospinal fluid can further increase CGRP, and thus worsen migraine [20]. Similarly, in states of neuroinflammation, abnormal cerebrospinal fluid flow can also enable reactive oxygen species to accumulate, triggering more inflammation, and thus inciting more migraines [20]. It is well known that disruption to arachnoid or Pacchionian granulation function or structure causes headaches, often through meningeal irritation or abnormal cerebrospinal flow dynamics [22]. Therefore, the glymphatic system is another consideration in the pathogenesis of migraines.

Beyond aberrations in carotid, cerebral, and meningeal vascular structure, and function in the context of migraine, systemic vasculopathy has been well documented among migraine patients. On the one hand, it has been shown that migraine, including MA and MO, is associated with an increased risk of myocardial infarction (HR 1.39) and cardiovascular mortality (HR 1.37), and cardiovascular diseases such as hypertension and ischemic heart disease are more frequent in migraine patients [23]. Another large study featuring 394,942 people with migraines and 757,465 people without migraines found that migraine was associated with a higher risk of major adverse cardiovascular and cerebrovascular events [24]. 

In terms of additional mediators of a vascular link to migraine, some have postulated hypercoagulability through the clotting cascade as a factor in migraine. There have been theories about platelet count and platelet function being involved with migraine, with platelet clots being a source of microemboli to cause the transient ischemia related to migraine, but there have been no significant studies to date [25,26]. In a 2018 case–control study, migraineurs had significantly elevated fibrinogen (316.12 mg/dL vs. 298.87 mg/dL, *p* = 0.007) and high-sensitivity CRP (3.42 mg/L vs. 2.56 mg/L, *p* = 0.03) [27]. Interestingly, the biomarker levels correlated with aura frequency and total number of aura-years. Other intravascular procoagulant factors that have similarly been found to be elevated include antiphospholipid antibody, homocysteine, von Willebrand factor, and endothelin-1, which all increase hypercoagulability and vasoconstriction [28,29,30]. Although migraine pathophysiology has much yet to be elucidated, migraine is increasingly recognized as a neurovascular disorder. 

Further demonstrating the link between vasculopathy and migraine is the correlation between different genetic mutations and migraine. Cerebral Autosomal-Dominant Arteriopathy with Subcortical Infarcts and Leukoencephalopathy (CADASIL) is a disease of the small vessels of the brain due to aberrant smooth muscle secondary to a mutation of the gene Notch3. Migraine, specifically MA, is a common complaint of people with CADASIL even before they have ischemic strokes related to their arteriopathy, with atypical features like prolonged aura and duration of deficits to resemble migrainous infarcts [31]. As the imaging patterns of white matter hyperintensities due to small vessel involvement in both migraine and CADASIL are similar, it may be the disease of these vessels that is a pathological driver of the symptoms of migraine, with MA reflecting temporary ischemia and temporary symptoms, whereas lacunar strokes, the most common type of stroke in CADASIL, are more permanent symptoms [31]. Interestingly, mouse models of CADASIL have shown increased susceptibility to CSD, revealing the mechanistic connection between CADASIL and migraine [32]. Other genetic conditions and the associated genes related to migraine and stroke include Retinal Vasculopathy with Cerebral Leukodystrophy (RVCL) from a mutation of TREX1, Mitochondrial myopathy with Encephalopathy, Lactic Acidosis and Stroke-like episodes (MELAS) from the MT-TL1 mutation, and other fibrovascular genetic conditions [33]. Patients with MELAS often develop migraines, and these migraines prove to be intractable. In fact, there is a case report of a person with MELAS presenting with MA refractory to triptans with imaging confirming infarction who later required erenumab to prevent migraines after trials of many prophylactic medications, highlighting another link between migraine and stroke and how migraine medications can still be employed in complex conditions like MELAS that can involve stroke-like episodes [34]. Table 1 summarizes the risk factors for stroke for people with migraine that have been discussed.

## 4. Ischemic Stroke

The underpinnings of blood vessels in migraine propagation have long called attention to the similarities between stroke and migraine. Aura is associated with roughly 30% of migraines, and its acute visual, sensory, or speech symptoms can be mistaken for ischemic stroke [35]. Features of aura that are distinct from stroke include multiple symptoms occurring in succession, as opposed to simultaneously, and symptoms crossing midline. 

The relative risk of ischemic stroke is well established to be approximately double in people with MA, compared to those with neither migraine nor MO [36,37,38,39,40]. The risk of ischemic stroke in MO has not been established [41]. Despite the increased risk, the prevalence of ischemic stroke in patients with migraine remains relatively low. It was estimated to be 1.3% in a national retrospective cohort study of more than 800,000 young adults with all migraine [36]. 

In the Women’s Health Study, the risk of ischemic stroke was still significant after adjusting for additional stroke risk factors such as smoking, exercise, body mass index, diabetes, and high cholesterol [23,41]. This suggests an independent vascular risk in MA. The risk of ischemic stroke may be highest among women aged 25–45 years (RR = 1.7), oral contraceptive users, and smokers [36,41]. There is an increased risk in active MA, defined as an attack within the past year, but not with longer duration of attack or higher frequency of attacks [36,41]. The risk in MO is less certain than that in MA but may be increased as well (OR/RR 1.34, *p* < 0.0001) [41]. The risk of ischemic stroke in men with migraines is not well established, but large pooled studies demonstrate a clear pattern. A meta-analysis of 11 case–control studies and 3 cohort studies published before 2004 showed that, relative to individuals without migraine, the risk of stroke was increased in migraineurs (pooled relative risk [RR], 2.16; 95% confidence interval [CI], 1.9–2.5). This risk was higher for those with MA (RR, 2.27; 95% CI, 1.61–3.19), but was also apparent in patients with MO (RR, 1.83; 95% CI, 1.06–3.15) [42].

## 5. Hemorrhagic Stroke

There may be an increased risk of hemorrhagic stroke in migraineurs, but this risk has not been as consistently demonstrated as it has in ischemic stroke. A 2013 meta-analysis of eight studies including 1600 hemorrhagic strokes found elevated risk in subjects with any migraine compared to controls (RR = 1.48, *p* = 0.002), and that this risk may be higher in female migraineurs less than 45 years old (1.57, *p* = 0.012) [43]. Other studies have found no association of hemorrhagic stroke with migraine, with or without aura [44], but other studies suggest a slightly higher risk for hemorrhagic stroke for people with MA [31,45]. There is a relatively low prevalence of hemorrhagic stroke in migraine. In 13 years of follow-up of participants in the Women’s Health Study, 9 out of 1435 (0.63%) with aura and 3 out of 2177 (0.14%) without aura developed a hemorrhagic stroke [45]. However, the risk for intracerebral hemorrhage was increased in women with active migraine with aura (2.25, 1.11 to 4.54, *p* = 0.024) and not increased in those with MO [45]. So, there does appear to be an increased risk of hemorrhagic stroke in MA.

## 6. Migrainous Infarction

Certain authors have attempted to generate a separate list of underlying factors at play for migrainous infarction and non-migrainous infarction occurring in patients with migraines [46]. The main clinical distinction between these two entities as per the third edition of the International Classification of Headache Disorders (ICHD-3) is that the former occurs during a migraine attack, but the latter occurs in an inter-ictal period [47]. The principal diagnostic differentiator between migrainous and non-migrainous infarction is that the migrainous infarction must also have evidence of an acute infarct on neuroimaging to clinically correspond with the aura symptoms experienced and have symptoms that last more than 60 min, whereas non-migrainous infarction does not have to correlate to typical aura or migraine symptoms. Furthermore, imaging studies such as magnetic resonance imaging (MRI) can demonstrate evidence of acute ischemic changes in anatomically relevant areas in the context of migrainous infarction, another similarity between typical ischemic stroke and strokes related to migraine [48]. Conversely, migraine or aura symptoms not causing concomitant infarction would not demonstrate evidence of acute ischemia on imaging.

Imaging is therefore an important part of the diagnostic process for migraine and stroke. Classic imaging findings in chronic migraine on MRI are deep white matter T2 hyperintensities, and even infratentorial hyperintensities and infarct-like lesions more typically in the posterior circulation in the setting of MA [13]. In fact, a cross-sectional study demonstrated a clear increased risk for white matter hyperintensities and subclinical lesions in people with migraine compared to people without migraine [49]. So, migraine is a certain risk factor for white matter changes seen on MRI. However, migraine is only one risk factor for white matter hyperintensities, and more common risk factors are hypertension, vascular disease, and aging [50]. These T2 hyperintensities can be differentiated from ischemic changes related to an acute stroke based on the presence of overlapping diffusion-weighted imaging and apparent diffusion coefficient signals in the setting of acute stroke. There are different theories for the chronic imaging findings such as vasoconstriction from ergotamines or other treatments causing small vessel ischemic changes in the form of white matter hyperintensities and even alterations in resting cerebral blood flow [33]. However, as ergotamine use is less common now, and as triptans can cause reversible cerebral vasoconstriction syndrome, there may be a small, inconsistent intracerebral vasoconstriction induced by triptans which contributes to white matter changes seen on MRI. Thus, medications as a cause of imaging findings in patients with migraine appears to be a tenuous explanation and it is likely the underlying pathophysiology of migraine and changes in blood flow dynamics causing chronic imaging changes. 

### Shared Cardiovascular Risk Factors of Migraine with Aura and Ischemic Stroke

The complexity of the relationship between migraine and stroke remains more fluid at present, and migraine patients who experience stroke may have components of both particularities of migraine itself predisposing to stroke and factors which may not be seemingly directly related to migraine, such as atrial fibrillation and vascular disease. Atrial fibrillation is an established risk factor for ischemic stroke, specifically cardioembolic stroke [51]. Multiple studies and even systematic reviews evaluating population-level data observe a greater frequency of atrial fibrillation in people who have migraine with aura compared to patients who experience without aura, and atrial fibrillation is observed as a more common risk factor in people with migraine with aura who have strokes [52,53]. For people with migraine with aura who develop strokes, cardioembolic stroke was the most common underlying mechanism, especially in people who experience visual auras [54]. Among stroke patients who do not have an aura associated with their migraines, they tend to have more common stroke risk factors like atherosclerosis [52]. Interestingly, having a patent foramen ovale (PFO) was also associated with stroke in those who have migraine with auras [52]. A review of several studies found that, among migraine patients, the incidence of PFO is 46.3–88% in migraine patients with aura compared with 16.2–34.9% in migraine patients without aura. Those who have migraine without aura do not appear to have a significantly higher prevalence of PFO in comparison to the general population [55]. Therefore, having a PFO is a risk factor for both migraine with aura and for stroke. This finding, along with other data, further demonstrates how embolism causing temporary ischemia, a common theory behind the negative symptoms associated with an aura, is implicated in both migraine with aura for temporary symptoms and migrainous infarction when negative symptoms persist because of stroke [53,54,56]. To provide more mechanistic insight, a mouse model revealed that microembolism induces CSD, revealing that the principal pathologic process of MA is triggered by emboli [57]. This association between embolism and migraine with aura, along with the certain link between embolism and stroke, further demonstrates the relationship between migraine with aura and stroke, and why migraine without aura is less associated with stroke or migrainous infarction.

Due to the known alterations in the size of blood vessels during migraines, oligemia and vasospasm could be factors in migrainous infarction. The driver of oligemia, or aberrant blood flow in the context of migraines, is cortical spreading depression as changes in brain activity lead to proportional changes in cerebral blood flow because of shifting metabolic demand. As these alterations in cortical activity do not respect vascular territories, there are many vessels involved in the subsequent oligemia. Therefore, oligemia is not a likely cause of infarction unless a patient is already predisposed, such as from a genetic condition that alters many blood vessels. In fact, most ischemic lesions in the context of migraine only involve one vessel. This indicates that unless someone has a pre-existing significant stenosis or abnormal vasculature, changes in blood flow are only capable of causing a stroke if there is a structural abnormality rather than a dynamic flow abnormality. However, this primarily applies to large vessel strokes, as the genetic small vessel disorders and imaging findings of small vessel disease demonstrate that flow dynamics of small vessels are a separate pathological process of migrainous infarction if there is a lacunar infarct.

## 7. Treatment 

Acute migraines can be treated in a variety of ways. Ergotamines, which are currently far less prescribed for the acute treatment of migraine, have been used since 1926 and were the only specific abortive migraine medication available for decades [58]. Their anti-migraine effect is thought to be achieved by inducing vasoconstriction through 5HT-1-B and 5HT-1D receptors on extracranial blood vessels. They also activate other 5-HT1 subtypes, dopamine, and noradrenaline receptors, which are responsible for side effects including hypertension and vasoconstriction [59]. Due to their vasoconstrictor effect, ergotamines are contraindicated in coronary artery disease, stroke, uncontrolled hypertension, and peripheral vascular disorders. A 2014 systemic review of observational studies investigating cardiovascular risk found that the intense consumption of ergotamines may be associated with an increased risk of serious ischemic complications (OR 2.28, 95% CI 1.18–4.41) [60]. Therefore, in current clinical practice, ergotamines have fallen out of favor with the development of alternative medications. 

Triptans are a class of abortive migraine medications with a more targeted mechanism of action and a somewhat better safety profile than ergotamines. They act as selective agonists toward serotonin 5HT-1B and 5HT-1D receptors on extracranial blood vessels innervated by the sensory nerves of the trigeminal system, causing vasoconstriction and inhibiting the release of pro-inflammatory neuropeptides by trigeminal terminals and thus nociceptive transmission [61]. Like ergotamines, their mechanism of action theoretically contraindicates use in patients with ischemic heart disease, history of myocardial infarction (MI), or stroke, and use is cautioned in patients with vascular risk factors. However, triptan treatment has not been shown in studies to consistently increase the risk of stroke. A 2004 observational cohort study of 63,575 migraine patients found no association between triptan prescription and stroke (HR 1.13, CI 0.78–1.75) or MI (HR 0.93, 95% CI 0.60–1.43) [62]. In a 2024 case-crossover study of 429,612 individuals, triptan prescription redemption was associated with a higher risk of ischemic events (MI OR 3.3 95% CI 1.0–10.9, ischemic stroke OR 3.2 CI 1.3–8.1), but case patients had a median age of 60 years old with a high-risk cardiovascular profile [63]. For patients with low cardiovascular risk, the risk of an ischemic event after triptan initiation was very low, and the study concluded that risk for individual users was very low. 

The development of newer migraine drugs has sought to expand safe and effective treatment options with drugs with non-vasoconstricting mechanisms of actions. Lasmiditan selectively targets 5-HT1F inhibitory receptors on central and peripheral trigeminal neurons, decreasing neuropeptide release and inhibiting pain pathways [64]. There was no statistical difference in the frequency of cardiovascular treatment-emergent adverse effects in its phase three trial [64]. Calcitonin gene-related peptide (CGRP) blockade is another new development in migraine treatment, which works through receptor or molecule antagonism. CGRP is a neuropeptide released from perivascular nerve fibers after trigeminal nerve activation [65]. Animal models demonstrate a vasodilatory effect of CGRP, which is likely a way it is implicated in migraine [66]. Anti-CGRP drugs have not been shown to have an increased risk of adverse cerebrovascular events either [67,68]. Furthermore, erenumab and fremanezumab are monoclonal antibodies that block CGRP and are effective for migraine prophylaxis. While there are mixed data on erenumab-induced hypertension, with most indications suggesting a slight increase in blood pressure, there were no clinical vascular adverse effects in a combined review of the clinical trial data [69,70,71,72,73]. 

Migrainous infarction is a rare subset of stroke with proposed pathophysiology of CSD, vasoconstriction, and ischemia, but there is no uniform treatment [74]. In general, experts recommend avoiding vasoconstrictive medications like ergotamines and triptans, and many alternative therapies have included nitrate, nifedipine, furosemide, valproate, acetazolamide, ketamine, prochlorperazine, and magnesium [74]. Some advocate for the treatment of migrainous infarction with methylprednisolone and hydration and even calcium channel blockers such as verapamil to reduce vasospasm and facilitate perfusion, along with the avoidance of triptans due to associated vasoconstriction [75]. The use of calcium channel blockers in this clinical scenario further demonstrates the importance of blood vessel dynamics rather than thrombosis in migrainous infarction.

## 8. Future Directions

Promising efforts in identifying migraine biomarkers have been made towards the goal of improving the diagnosis and treatment of migraine. CGRP, for example, is considered the initiative biomarker, as it was found to have significantly increased levels in peripheral blood during the interictal period, which led to the development of CGRP-targeted therapy [76]. However, despite this, there are still no validated biomarkers for migraine [77,78]. 

MicroRNA (miRNA) is one such area that is being studied in multiple applications related to migraine. miRNA are short, non-coding RNAs that control messenger RNA expression and inhibit the function of proteins responsible for the development of migraine pain through epigenetic mechanisms [79]. They have been shown to be deregulated in pain states and diseases associated with increased cardiovascular risk, which identifies its potential as a biomarker and may help further clarify the relationship between migraine and stroke [77]. A specific circulating miRNA profile was found to be associated with migraine without aura which is also known to be modulated in atherosclerosis and stroke [80]. Another study found the similarly elevated expression of four miRNAs that regulate endothelial function in migraine patients with aura compared to those without aura [81]. These preliminary studies could suggest a link between migraine without aura and cardiovascular risk. Future studies are needed to investigate the complex link between migraine, microRNA, and vascular risk further. Other peptides being evaluated in the context of migraine are secretin peptides such as pituitary adenylate cyclase-activating polypeptide (PACAP) and vasoactive intestinal peptide (VIP), and to a lesser extent, other peptides in the same class [82]. The data appear to be more mixed for VIP than PACAP. Levels of PACAP are increased in blood tests during migraine; infusing PACAP triggers migraines through vasodilation, neurogenic inflammation, and nociception; and there is some data indicating that blocking the PACAP pathway can reduce monthly migraine burden [82]. Therefore, serum PACAP levels can serve as a biomarker for migraine, while medications targeting PACAP or its ligand can serve as therapeutic targets, with clinical trials underway [82].

## 9. Limitations

There are limitations to the methods employed in this scoping review such as the incomplete retrieval of identified research and reporting bias. The restrictions imposed on the search strategy to exclude publications that were not in English, did not have a full text available, or that were clinical trials, case series, or case reports led to a significant decrease in the studies available for review. The date range of publications available on the PubMed database from 1976 to 2024, while comprehensive, does exclude many earlier studies that elucidate the pathophysiology of migraine, so the nature of using an online database, which only features electronically available studies, is a limitation. Moreover, analysis often combined conclusions from different studies to explain different components of the relationship between migraine and stroke, inviting conflation and mixed findings. The broad methodology and preference for population-level clinical data was a method to account for these limitations along with the inclusion of negative studies to display the complex link between these two conditions.

## 10. Conclusions

This scoping review reveals the connection between migraine and stroke in terms of risk factors, mechanistic pathophysiology, clinical presentation, and management. Over time, the pathophysiology of migraine has come to be understood to be complex. There is an increased risk of ischemic stroke in patients with migraine with aura, but the prevalence remains relatively low. The risk of ischemic stroke is not well established in migraine without aura. Less certain is the risk of hemorrhagic stroke in all migraines. While initial migraine-targeted therapies work by vasoconstriction and are contraindicated in patients with vascular risk factors or history, the risk for individual users appears to be relatively low. Translational advances have allowed for the development of newer therapies that provide safe and effective therapies for all who suffer from migraine.

## Figures and Tables

**Table 1:** Risk factors for stroke in migraine.

Excessive Vasodilation
Cortical Spreading Depression
Cerebral Hypoperfusion
Oligemia
Endothelial Dysfunction or Structural Vasculopathy
Atrial Fibrillation (cardioembolic) *
Patent Foramen Ovale (paradoxical embolism) *
Microembolism (thromboembolism) *
Hypercoagulability
MELAS
Inflammation

* Only migraine with aura due to microemboli, not related to risk of migraine without aura.
